# Efficacy and Safety of Intradermal Versus Subdermal Injection of Polynucleotide for Facial Improvement: A Prospective Split‐Face Study

**DOI:** 10.1111/jocd.71108

**Published:** 2026-08-04

**Authors:** Hyungkyu Bae, Soo‐Bin Kim, Yu‐Ran Heo, Hyoun Jun Park, Jong Seo Kim, Nark‐Kyoung Rho, Hee‐Jin Kim

**Affiliations:** ^1^ Department of Anatomy Yonsei University Wonju College of Medicine Wonju Republic of Korea; ^2^ Department of Oral Anatomy, Institute of Biomaterial Implant, College of Dentistry Wonkwang University Iksan Republic of Korea; ^3^ Division in Anatomy and Developmental Biology, Department of Oral Biology, Human Identification Research Institute, BK21 FOUR Project Yonsei University College of Dentistry Seoul Republic of Korea; ^4^ Maylin Clinic Seoul Republic of Korea; ^5^ Kim‐Jongseo Plastic Surgery Clinic Seoul Republic of Korea; ^6^ Leaders Aesthetic Laser and Cosmetic Surgery Center Seoul Republic of Korea

## Abstract

**Background:**

Polynucleotide‐based injectables are increasingly used to improve periocular and midface skin quality, but the optimal delivery approach remains uncertain. Because intradermal and subdermal administration differ in tissue placement and short‐term procedure‐related reactions, clarifying whether subdermal delivery can achieve comparable objective improvement with better tolerability is clinically relevant.

**Aims:**

This study aimed to compare the efficacy and safety of intradermal versus subdermal delivery of polynucleotide for facial skin improvement using a prospective split‐face clinical design.

**Patients/Methods:**

In this prospective split‐face study, adults seeking facial wrinkle improvement received three treatment sessions at 3‐week intervals. The left cheek received intradermal injection and the right cheek received subdermal delivery. The primary endpoint was percentage improvement from baseline to Week 12 in the Antera 3D fineline index. A prespecified non‐inferiority margin of −15% (SD − ID) was evaluated using baseline‐adjusted linear mixed‐effects modeling. Secondary objective outcomes included additional Antera 3D skin‐quality indices and skin elasticity (Cutometer R2). GAIS, post‐procedure pain (VAS), injection‐site embossing, and adverse events were recorded.

**Results:**

Forty participants received at least one treatment, and 37 contributed imaging‐based efficacy data. Both approaches improved wrinkle‐ and texture‐related parameters from baseline. Subdermal delivery met the non‐inferiority criterion for fineline improvement at Week 12 and also showed non‐inferior results at Week 9. At Week 12, the fineline index was lower on the subdermal side than on the intradermal side. Subdermal delivery was associated with lower pain scores at all sessions and no injection‐site embossing.

**Conclusions:**

Subdermal delivery was non‐inferior to intradermal injection for objective skin‐quality outcomes and demonstrated a more favorable tolerability profile.

## 1 Introduction

Facial skin aging is a multidimensional process that manifests as fine wrinkles, textural irregularities, loss of elasticity, and uneven tone [1, 2, 3, 4]. These changes drive demand for minimally invasive interventions that aim to improve global “skin quality” outcomes with acceptable tolerability and minimal downtime [5, 6, 7, 8, 9, 10].

Injectable aesthetic treatments have expanded beyond volumetric correction to include approaches targeting subtle, surface‐level changes in skin appearance and biomechanical properties [11, 12]. Clinical studies of injectable fillers, including split‐face designs, illustrate the feasibility of assessing nuanced aesthetic outcomes and underscore the importance of standardized technique and safety monitoring in facial injection practice [13, 14, 15, 16, 17, 18].

Polynucleotide (PN)–based injectable medical devices have been increasingly used in aesthetic practice as skin‐quality–oriented interventions. Published clinical reports and trials have evaluated PN‐based products in periocular indications, including crow's feet correction, with generally favorable effectiveness and safety profiles reported under controlled study conditions [17, 19, 20, 21, 22]. Nevertheless, across the available literature, treatment protocols vary widely in injection technique, anatomical targets, and endpoints, which limits procedural standardization and makes it difficult to infer the optimal technique for consistent, reproducible outcomes, including the optimal injection plane [20, 23].

Among procedural parameters, injection plane is a clinically important yet insufficiently resolved variable. The major published protocols and consensus recommendations for PN‐based skin rejuvenation have predominantly described intradermal delivery, typically using needle‐based microdroplet, serial puncture, or linear retrograde techniques [17, 19, 20, 23, 24]. However, intradermal delivery can be technically demanding and may be associated with greater procedural pain and transient visible surface irregularities [20, 23, 25]. In contrast, controlled evidence directly comparing intradermal and subdermal PN delivery using the same product and the same anatomic treatment field remains limited. Therefore, a prospective split‐face design provides a robust framework to evaluate technique‐dependent differences because each participant serves as their own control, thereby minimizing inter‐individual variability [13, 16, 25].

Objective, instrument‐based assessment is particularly valuable in skin‐quality trials where expected changes can be subtle and multidimensional. Three‐dimensional imaging systems quantify parameters related to skin surface and tone, and Antera‐based indices have been evaluated for wrinkle/roughness and pore assessment as well as erythema‐ and melanin‐related measurements against established instruments, supporting their use as quantitative endpoints when imaging is standardized [26, 27, 28, 29, 30, 31]. PN clinical studies have also adopted three‐dimensional imaging endpoints to complement investigator‐ and subject‐reported assessments in facial aesthetic settings [17, 19].

Therefore, the present prospective split‐face clinical trial was designed to compare the efficacy and safety of intradermal versus subdermal injection of a PN‐based injectable medical device for infraorbital cheek skin‐quality improvement using standardized technique and objective instrument‐based outcomes, thereby informing more evidence‐based technique selection in clinical practice.

## 2 Materials and Methods

The study was conducted in accordance with the Declaration of Helsinki and was approved by the Public Institutional Review Board designated by the Ministry of Health and Welfare of the Republic of Korea (approval No. P01‐202404‐01‐049).

### 2.1 Study Design

This prospective split‐face clinical study compared the efficacy and safety of intradermal versus subdermal injection of a polynucleotide‐based injectable for facial skin improvement. In all participants, the left cheek was treated intradermally and the right cheek was treated subdermally. Treatments were administered at 3‐week intervals for three sessions, with follow‐up assessments at Weeks 9 and 12.

### 2.2 Participants

Adults aged ≥ 19 years who sought improvement of facial wrinkles were eligible. Participants were required to agree to discontinue any facial dermatologic or aesthetic procedures aimed at wrinkle improvement during the study period and to provide written informed consent. Exclusion criteria included facial filler injection within the previous 6 months; current use of NSAIDs, antiplatelet agents, anticoagulants, or immunosuppressants; allergic, autoimmune, or granulomatous disease (including sarcoidosis); hypersensitivity to sodium polynucleotide; pregnancy or breastfeeding; inability to provide informed consent; and active inflammation or infection at the treatment sites. Full eligibility criteria are provided in Table S1.

### 2.3 Injection Procedure and Plane Confirmation

**FIGURE 1:**
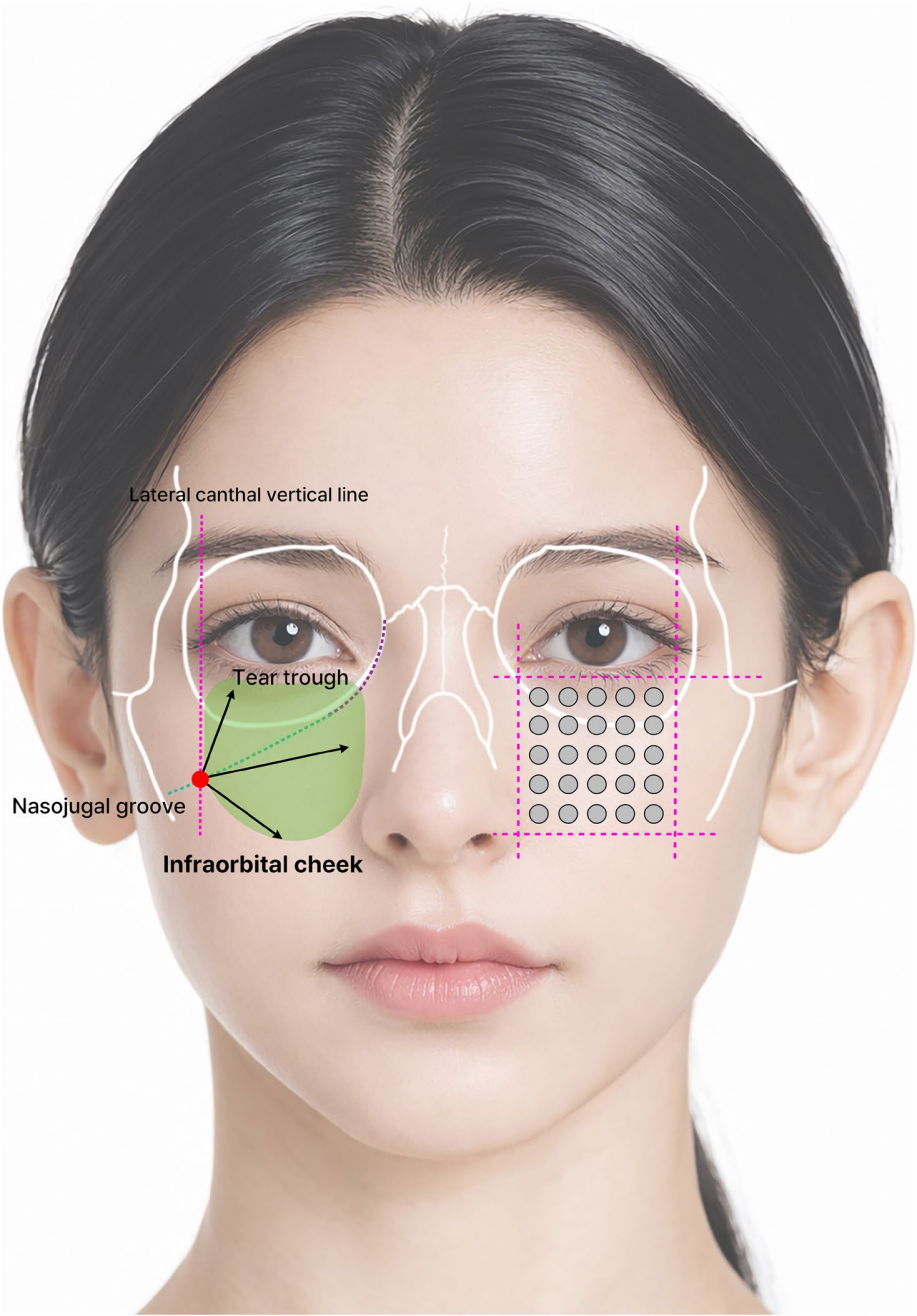
Injection protocol and treatment area for periocular/infraorbital skin improvement. Schematic illustration of the standardized injection plan. The infraorbital cheek treatment field (green shading) is shown adjacent to the tear trough and nasojugal groove. The magenta dotted lines indicate the anatomical reference boundaries used to standardize the treatment area, defined by the medial and lateral canthal vertical lines, a horizontal line passing through the nasal ala, and the inferior eyelid margin. Open circles denote the predefined intradermal serial puncture points used for the ID side. For the SD side, product was delivered via a cannula introduced at the intersection of the nasojugal groove and the lateral canthal vertical reference line and distributed within the same predefined zone.

All treatments were performed according to a standardized, anatomically bounded protocol for the infraorbital cheek (Figure 1). The treatment field was defined using the medial and lateral canthal vertical lines, a horizontal line passing through the nasal ala, and the inferior eyelid margin, and was applied consistently across visits. Topical anesthetic cream was applied for at least 30 min before injection. Rejuran (sodium polynucleotide; PharmaResearch Co. Ltd., Republic of Korea) was administered to each cheek at each session (maximum 1 mL per cheek; 2 mL per session). In all participants, the left cheek was treated intradermally (ID) using a serial puncture technique, placing multiple intradermal deposits at predefined grid points within the designated zone. The right cheek was treated subdermally (SD) using a cannula‐based technique: the cannula was introduced at the intersection of the nasojugal groove and the lateral canthal vertical reference line, and the product was distributed within the predefined infraorbital‐cheek zone in the subdermal plane. Injection plane was confirmed by ultrasound immediately before and after treatment with direct visualization of the dermal layer. Procedures were performed by three investigators using the same standardized technique. A touch‐up procedure was permitted by protocol in case of clinically meaningful asymmetry; however, no participants required or received touch‐up treatment (0%), and all analyses were therefore conducted without additional intervention beyond the planned treatment sessions.

### 2.4 Outcome Measures

#### 2.4.1 Primary Outcome

The primary endpoint was the percentage improvement from baseline to Week 12 (i.e., 6 weeks after the final injection) in the fineline index, quantified from standardized Antera 3D imaging (Miravex Ltd., Dublin, Ireland) obtained separately for each cheek.

#### 2.4.2 Secondary Outcomes (Objective Skin‐Quality Measures)

Secondary outcomes included (1) additional outcomes derived from the same standardized Antera 3D imaging and (2) skin elasticity measured with Cutometer MPA580 (Courage + Khazaka electronic GmbH, Cologne, Germany; R2). Specifically, secondary outcomes comprised changes from baseline at Weeks 9 and 12 in:

1. Wrinkle index.
2. Roughness (texture).
3. Pore parameter.
4. Melanin‐related parameter.
5. Hemoglobin‐related parameters (hemoglobin index and hemoglobin variation).
6. Skin elasticity (Cutometer MPA580, R2)

#### 2.4.3 Supportive Outcomes

Supportive outcomes included Global Aesthetic Improvement Scale (GAIS) rated by both investigator and participant at follow‐up visits, post‐procedure pain assessed using a visual analog scale (VAS) within 10 min after each injection for each cheek, and injection‐site embossing graded as none or (if visible) mild/moderate/severe. Safety was assessed by monitoring adverse events throughout the study.

### 2.5 Statistical Analysis

Analyses were performed on a within‐subject basis consistent with the split‐face design. Continuous variables were summarized as mean ± standard deviation (or median with interquartile range, as appropriate), and categorical variables as counts and percentages. All analyses were conducted using IBM SPSS Statistics, version 27.0 (IBM Corp., Armonk, NY, USA).

The safety analysis set included all participants who received at least one treatment, and the efficacy analysis set included participants with at least one post‐baseline efficacy assessment. No imputation was performed for missing efficacy outcomes; analyses were based on available post‐baseline data from the efficacy analysis set.

The primary confirmatory analysis was the non‐inferiority analysis of percentage improvement in the Antera 3D fineline index from baseline to Week 12. The between‐side difference (SD − ID) and 95% confidence interval (CI) were estimated using a baseline‐adjusted linear mixed‐effects model with participant as a random effect to account for within‐subject pairing. Non‐inferiority was concluded if the lower bound of the 95% CI exceeded the prespecified −15% margin.

The non‐inferiority margin was prespecified as −15% (SD − ID) in percentage improvement, representing the largest clinically acceptable loss in fineline improvement for SD relative to ID. This margin was selected based on clinical judgment, general regulatory principles for non‐inferiority trial design, and precedent from aesthetic injectable/filler non‐inferiority trials in which 15% margins have been used for responder‐ or improvement‐rate endpoints [32, 33, 34]. Because SD delivery was expected to reduce immediate procedure‐related burden, including pain and visible embossing, a loss greater than 15 percentage points in objective fineline improvement was considered clinically unacceptable.

Sample size was calculated for the primary non‐inferiority endpoint in a paired split‐face design, with one‐sided *α* = 0.025 and 90% power. Because prior data directly comparing intradermal and subdermal PN delivery were unavailable, the calculation was based on planning assumptions of measurable improvement after the standardized three‐session PN protocol and no true between‐side difference. A minimum of 28 evaluable participants was required, and 40 participants were planned to allow for approximately 30% attrition.

Week 9 analyses of the primary endpoint were considered supportive. Analyses of secondary objective endpoints were exploratory, and no multiplicity adjustment was applied. Distributional assumptions were assessed using the Shapiro–Wilk test and visual inspection of the data. Paired *t*‐tests or Wilcoxon signed‐rank tests were used as descriptive/supportive analyses for observed within‐side or between‐side comparisons, according to distributional assumptions. Supportive categorical outcomes were evaluated using the chi‐square test or Fisher's exact test, as appropriate. A two‐sided *p*‐value < 0.05 was considered statistically significant for superiority‐type analyses where applicable.

## 3 Results

### 3.1 Patient Characteristics and Analysis Sets

**FIGURE 2:**
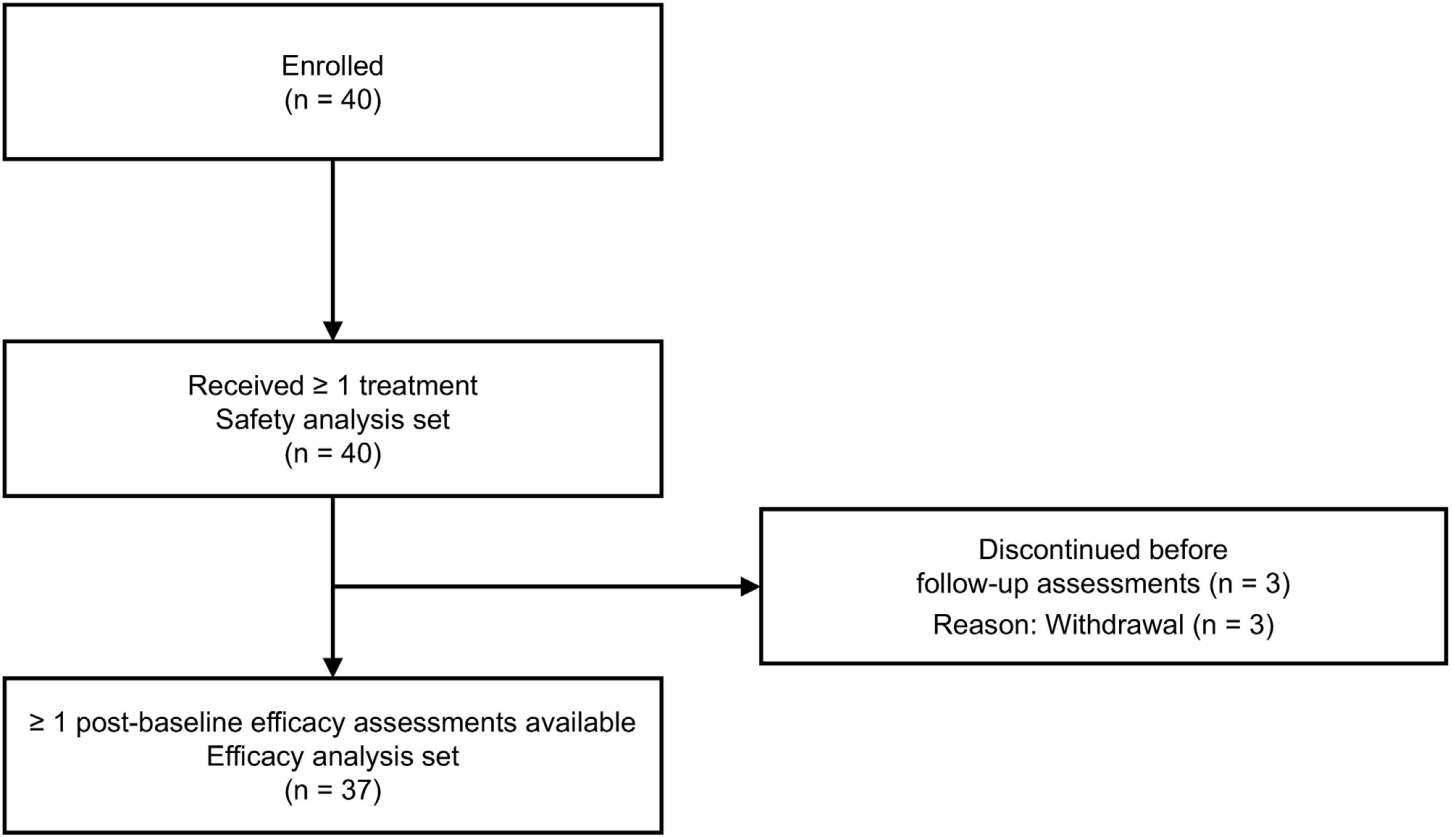
Participant disposition and analysis sets. A total of 40 participants were enrolled and received ≥ 1 treatment, comprising the safety analysis set (*n* = 40). Three participants discontinued before follow‐up assessments (reason: Withdrawal; *n* = 3). Participants with ≥ 1 post‐baseline efficacy assessment available were included in the efficacy analysis set (*n* = 37).

A total of 40 participants were enrolled and received at least one treatment, constituting the safety analysis set. The mean age was 31 ± 3.6 years; 3 participants (7.5%) were male and 37 (92.5%) were female. Three participants discontinued before follow‐up assessments (withdrawal), and 37 participants with at least one post‐baseline efficacy assessment were included in the efficacy analysis set (Figure 2). Baseline values for each outcome are presented in the corresponding tables; efficacy endpoints were analyzed using linear mixed‐effects models with baseline as a covariate to account for within‐subject pairing.

### 3.2 Fineline Index Over Time (Weeks 9 and 12)

**FIGURE 3:**
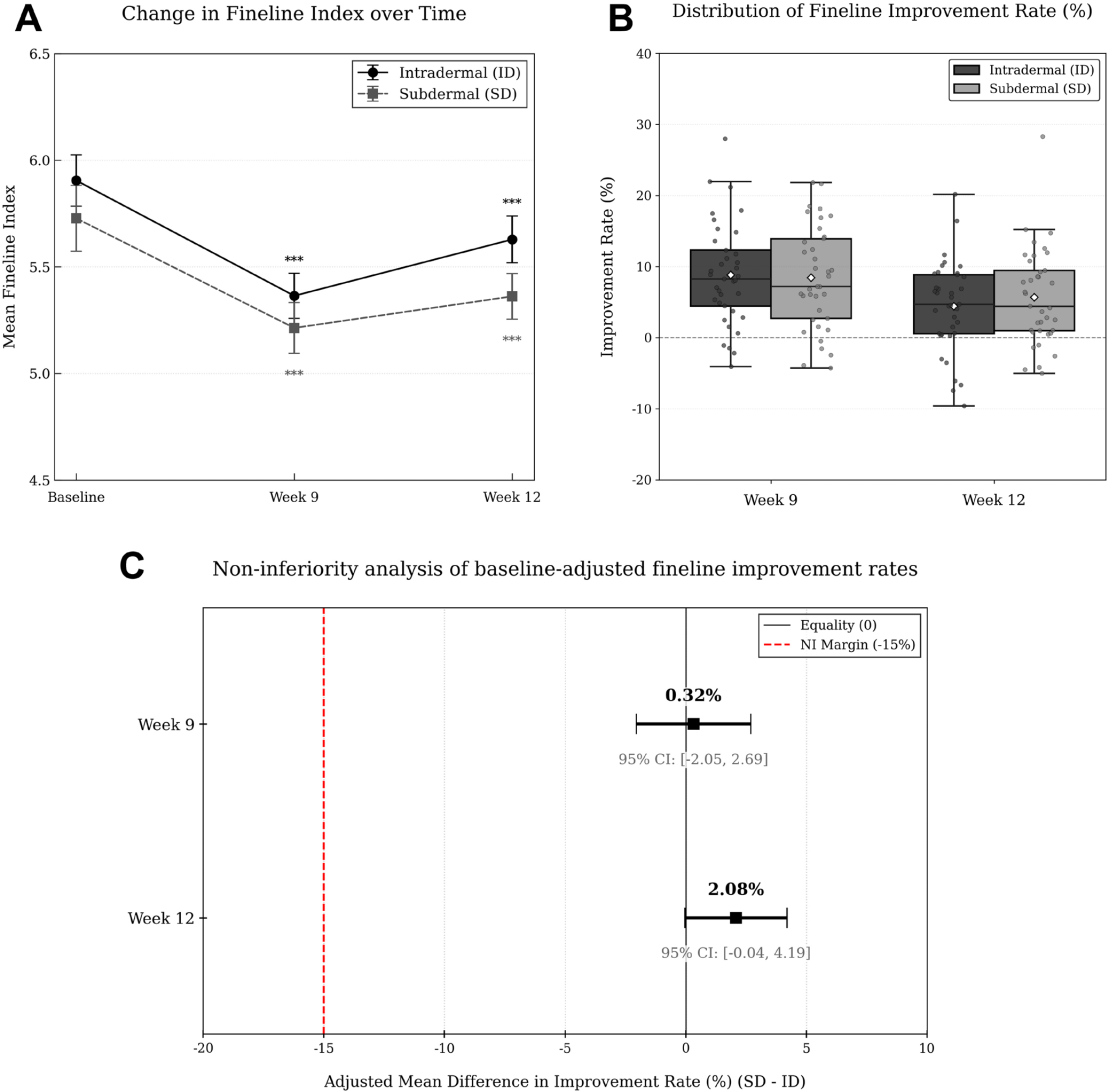
Fineline index outcomes (Weeks 9 and 12). (A) Mean Antera 3D fineline index values at baseline and at Weeks 9 and 12 for the intradermal (ID) and subdermal (SD) sides (*n* = 37). Error bars indicate the standard error of the mean (SEM). Asterisks denote within‐side change versus baseline (***p < 0.001). (B) Distribution of percent improvement from baseline in fineline index at Weeks 9 and 12 for the ID and SD sides (*n* = 37). Percent improvement was calculated as (baseline—follow up)/baseline × 100. (C) Baseline‐adjusted between‐side differences in percent improvement (SD − ID) at Weeks 9 and 12 estimated using a linear mixed‐effects model that accounted for within‐subject pairing with baseline as a covariate. Squares indicate adjusted mean differences and horizontal bars denote 95% CIs. The dashed vertical line indicates the prespecified non‐inferiority margin (−15%).

Both intradermal (ID) and subdermal (SD) injections produced improvement in the Antera 3D fineline index over time, with significant within‐side reductions from baseline at Weeks 9 and 12 on both sides (all *p* < 0.001; Table 1; Figure 3A). Mean fineline values decreased from baseline (ID: 5.90 ± 0.73; SD: 5.73 ± 0.94) to Week 9 (ID: 5.36 ± 0.64; SD: 5.21 ± 0.73) and remained improved at Week 12 (ID: 5.63 ± 0.67; SD: 5.34 ± 0.65). Between sides, fineline values did not differ significantly at baseline or Week 9, whereas at Week 12 the fineline index was lower on the SD side than on the ID side (*p* = 0.005; Table 1). Distributions of percentage improvement at Weeks 9 and 12 are shown in Figure 3B.

**TABLE 1:** Comparison of fineline index between intradermal and subdermal sides.

		Intradermal	Subdermal	*p*
Baseline	*N*	37	37	0.150^a^, ^c^
	Mean ± SD	5.90 ± 0.73	5.73 ± 0.94	
	[Min–Max]	[4.53–7.31]	[4.41–9.31]	
Week 9	*N*	37	37	
	Mean ± SD	5.36 ± 0.64	5.21 ± 0.73	
	[Min–Max]	[4.20–7.19]	[4.13–7.29]	
	*p*	**< 0.001** ^b^, ^c^	**< 0.001** ^b^, ^c^	0.126^a^, ^c^
Week 12	*N*	37	37	
	Mean ± SD	5.63 ± 0.67	5.34 ± 0.65	
	[Min–Max]	[4.52–7.56]	[4.37–6.87]	
	*p*	**< 0.001** ^b^, ^c^	**< 0.001** ^b^, ^d^	**0.005** ^a^, ^c^

*Note:* Values are presented as mean ± standard deviation and [minimum–maximum]. Bold values indicate statistical significance (*p* < 0.05).

Abbreviations: ID, intradermal; SD, subdermal.

^a^ Between‐side comparison between ID and SD.

^b^ Within‐side comparison versus baseline (0 W).

^c^ Analyzed by Paired *t*‐test.

^d^ Analyzed by Wilcoxon signed‐rank test (normality not met).

In baseline‐adjusted non‐inferiority analyses using the prespecified margin of −15% for the between‐side difference in percentage improvement (SD − ID), SD met the non‐inferiority criterion versus ID at both Week 9 and Week 12, with the lower bounds of the 95% CIs exceeding −15% (Week 9: −2.05%; Week 12: −0.04%; Figure 3C).

### 3.3 Secondary Objective Skin‐Quality Outcomes

Observed values for secondary objective outcomes (wrinkle index, roughness, pore parameter, melanin variation, hemoglobin index, hemoglobin variation, and skin elasticity [R2]) at baseline, Week 9, and Week 12 are summarized in Tables S2–S8. Longitudinal time‐course plots (mean ± SEM) are provided in Figure S1, and distributions of percent improvement at Weeks 9 and 12 are shown in Figure S2. Across secondary objective measures, both injection planes generally demonstrated improvement from baseline by Week 9, with maintenance of improvement through Week 12 for several domains, while other domains showed greater interindividual variability.

**FIGURE 4:**
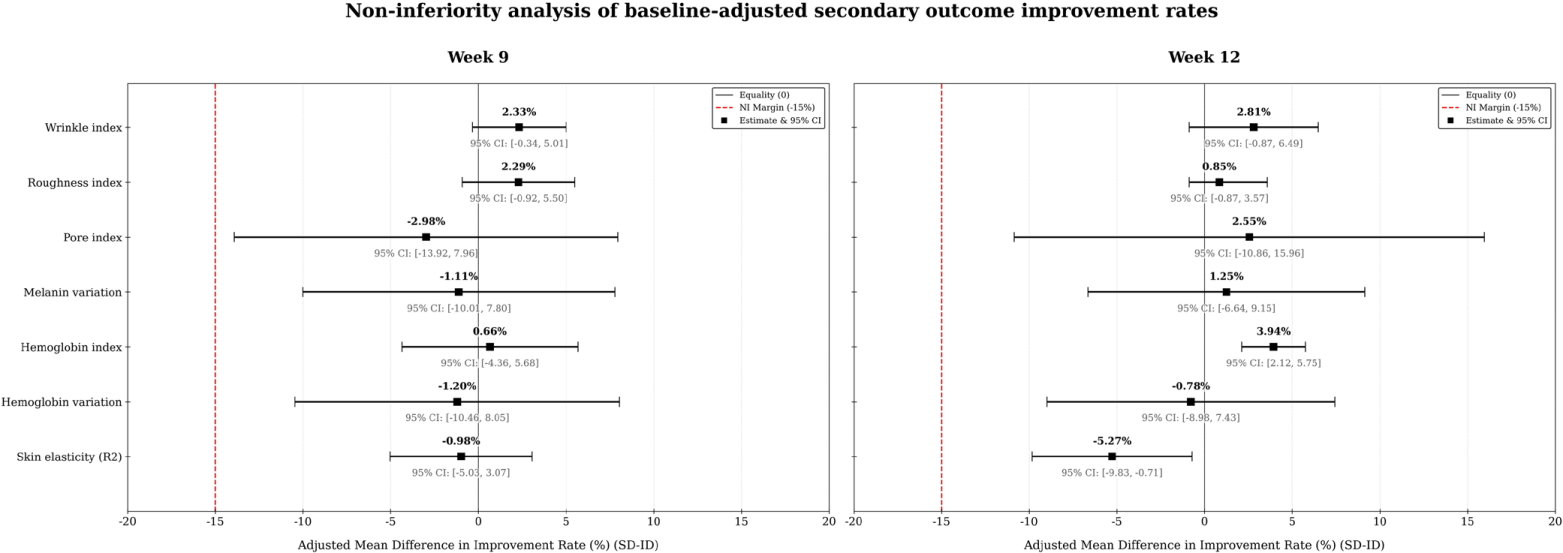
Exploratory baseline‐adjusted analyses of secondary outcome improvement rates using the prespecified non‐inferiority margin. Forest plots show baseline‐adjusted between‐side differences in percent improvement (SD − ID) for secondary objective skin‐quality measures at Weeks 9 (left) and 12 (right). Point estimates (squares) and 95% CIs (horizontal bars) were obtained from linear mixed‐effects models accounting for within‐subject pairing with baseline as a covariate. The solid vertical line indicates equality (0), and the dashed red line indicates the prespecified non‐inferiority margin (−15%). Positive values favor the subdermal (SD) injection side. Outcomes include wrinkle index, roughness index, pore index, melanin variation, hemoglobin index, hemoglobin variation, and skin elasticity (Cutometer R2).

Exploratory baseline‐adjusted analyses for secondary outcomes are summarized in Figure 4, which displays the between‐side difference in percent improvement (SD − ID) with 95% CIs at Weeks 9 and 12 using the prespecified non‐inferiority margin as a reference threshold. Although the lower bounds of the 95% CIs exceeded −15% for all secondary endpoints at both time points, these analyses were exploratory and were not adjusted for multiplicity. Therefore, these findings should be interpreted descriptively as broadly comparable short‐term responses between injection planes rather than as confirmatory non‐inferiority results. Between‐side estimates were generally small in magnitude, and CIs crossed 0 for most outcomes. Hemoglobin index at Week 12 showed a positive between‐side estimate with a 95% CI not crossing 0, whereas skin elasticity (R2) at Week 12 showed a negative between‐side estimate (Figure 4).

### 3.4 Representative Antera 3D Imaging

**FIGURE 5:**
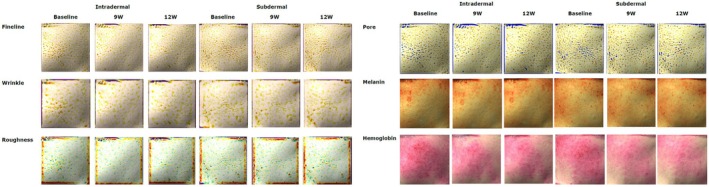
Representative Antera 3D maps. Representative Antera 3D output maps from a single participant are shown for fineline, wrinkle, and roughness (left panel) and for pore, melanin, and hemoglobin parameters (right panel) at baseline, Week 9, and Week 12 after intradermal injection (left cheek) and subdermal injection (right cheek). Images are provided to visually complement the quantitative analyses within the predefined infraorbital‐cheek treatment zone.

Representative Antera 3D maps illustrating fineline, wrinkle, roughness, pore, melanin, and hemoglobin parameters at baseline, Week 9, and Week 12 are shown in Figure 5. The representative images qualitatively demonstrate the spatial pattern and magnitude of changes within the predefined infraorbital‐cheek treatment zone.

### 3.5 Supportive Outcomes

#### 3.5.1 Global Aesthetic Improvement (GAIS)

Participant‐rated GAIS category distributions and responder rates (GAIS ≥ 1) at Weeks 9 and 12 are summarized in Figure S3 and Table S9. Investigator‐rated GAIS outcomes are summarized in Figure S3 and Table S10. Overall, both participant‐ and investigator‐rated assessments showed high proportions of “Improved” or better ratings on both sides at Weeks 9 and 12; these supportive results are presented descriptively (exploratory).

#### 3.5.2 Tolerability: Post‐Procedure Pain (VAS) and Injection‐Site Embossing

**FIGURE 6:**
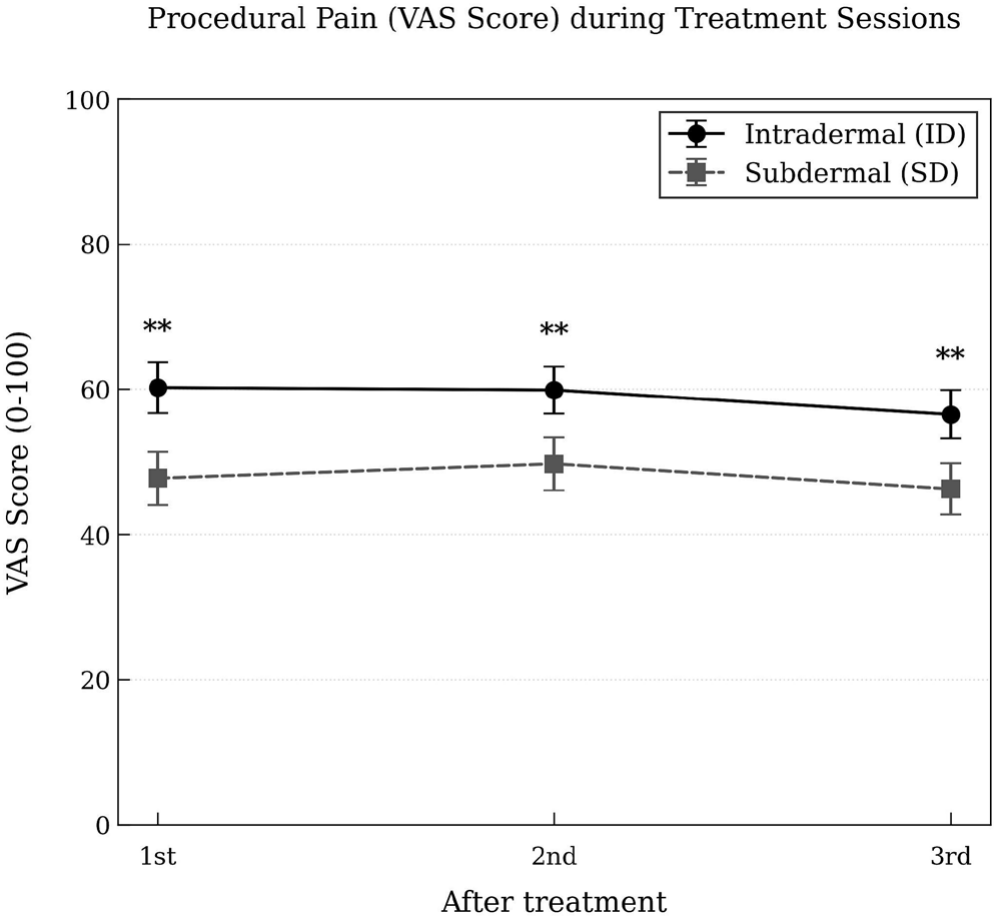
Procedural pain (VAS) over treatment sessions. Mean visual analog scale (VAS; 0–100 mm) pain scores assessed within 10 min after each treatment session are shown for the intradermal (ID) and subdermal (SD) sides (error bars, SEM). Asterisks indicate significant between‐side differences at the corresponding session (paired comparison; *p* < 0.01).

Procedural pain assessed using a 0–100 visual analog scale (VAS) after each treatment session was consistently lower on the SD side than on the ID side. Mean VAS scores (ID vs. SD) were 60.21 ± 21.33 vs. 47.73 ± 22.45 after the first session, 59.89 ± 19.56 vs. 49.70 ± 22.05 after the second session, and 56.54 ± 20.29 vs. 46.27 ± 21.50 after the third session, with statistically significant between‐side differences at all sessions (*p* < 0.01) (Table 2 and Figure 6).

**TABLE 2:** Comparison of procedural pain (VAS score) between intradermal and subdermal injection sides over time.

		Intradermal	Subdermal	*p*
Baseline	*N*	37	37	**0.004** ^a^
	Mean ± SD	60.21 ± 21.33	47.73 ± 22.45	
	[Min–Max]	[23–93]	[4–91]	
Week 3	*N*	37	37	**0.005** ^b^
	Mean ± SD	59.89 ± 19.56	49.70 ± 22.05	
	[Min–Max]	[19–99]	[16–99]	
Week 6	*N*	37	37	**0.004** ^a^
	Mean ± SD	56.54 ± 20.29	46.27 ± 21.50	
	[Min–Max]	[16–97]	[15–87]	

*Note:* Values are presented as mean ± standard deviation and [minimum–maximum]. Bold values indicate statistical significance (*p* < 0.05).

Abbreviations: ID, intradermal; SD, subdermal; VAS, visual analog scale.

^a^ Analyzed by paired *t*‐test.

^b^ Analyzed by Wilcoxon signed‐rank test (normality not met).

Injection‐site embossing was observed only on the ID side across treatment sessions, whereas no embossing occurred on the SD side at any session. On the ID side, embossing was predominantly graded as mild to moderate, with severe events occurring infrequently (Table S11).

## 4 Discussion

This prospective split‐face study evaluated whether injection plane—intradermal (ID) versus subdermal (SD)—influences objective skin‐quality outcomes and procedural tolerability following injection of a polynucleotide‐based injectable medical device [20]. These findings should be interpreted in the context of the existing PN literature, in which most published skin‐rejuvenation protocols have been based on intradermal delivery [17, 19, 20, 23]. The present study does not suggest that ID and SD injections have identical or universally interchangeable clinical roles. In practice, ID injection is commonly selected for superficial skin‐quality targets, whereas SD delivery may be considered for broader product spread, tissue support, or subtle volumetric effects. Because this study did not evaluate volumetric or structural support outcomes, the results should be interpreted within the scope of short‐term objective skin‐quality improvement. Within this scope, SD delivery achieved comparable objective improvement while reducing immediate procedure‐related pain and visible embossing, suggesting that SD injection may be a practical option when short‐term skin‐quality improvement and reduced peri‐procedural burden are prioritized [20, 23, 25].

Across objective imaging outcomes, both ID and SD injections produced measurable short‐term improvements in skin‐quality parameters. The primary non‐inferiority analysis demonstrated that SD injection was non‐inferior to ID injection for fineline improvement within the prespecified margin. Exploratory analyses of secondary Antera 3D–derived indices also showed broadly comparable responses between the two injection planes, although these findings should be interpreted cautiously because they were not adjusted for multiplicity. In contrast, tolerability outcomes demonstrated a clear procedural advantage for SD injection, with consistently lower post‐procedure pain and no injection‐site embossing on the SD side [25]. This finding is clinically relevant because conventional intradermal PN injection requires multiple superficial punctures and has been associated with procedure‐related discomfort and transient papules or embossing, even when topical anesthesia is used [25, 35]. At the same time, directional differences were observed for some parameters, suggesting that injection plane selection may be individualized based on the clinical target and patient characteristics.

Wrinkle‐ and surface‐related parameters, including fineline, wrinkle, and roughness indices, improved from baseline on both sides over time. For the primary endpoint, baseline‐adjusted between‐side estimates in percent improvement were directionally positive for SD at both Week 9 and Week 12 while meeting the prespecified non‐inferiority criterion at Week 12. However, because most between‐side differences in objective efficacy outcomes were small, these findings are best interpreted as indicating broadly comparable short‐term responses between the two injection planes rather than the superiority of one approach.

Pore‐related metrics also improved over time on both sides, but showed greater variability across individuals and time points than wrinkle‐ and roughness‐related measures. At both Week 9 and Week 12, between‐side estimates in percent improvement were small overall, and the direction of the estimate was not consistent across visits. This pattern is plausible for micro‐surface indices, which may be influenced by early post‐treatment tissue responses, imaging sensitivity, and acquisition conditions. Clinically, when pore‐related improvement is a primary treatment goal, plane selection may be individualized based on baseline skin characteristics and operator experience, while recognizing that pore parameters can exhibit more heterogeneous responses than other imaging‐derived indices [26].

Tone‐related outcomes required more cautious interpretation because these indices can be sensitive to baseline conditions and acquisition variability; therefore, baseline‐adjusted modeling was prioritized [31, 36]. For melanin variation, between‐side estimates were small overall and did not show a consistent directional pattern across Week 9 and Week 12. In contrast, hemoglobin‐related outcomes showed clearer separation after baseline adjustment: subdermal injection met the prespecified non‐inferiority criterion at both visits, and for the hemoglobin index, the between‐side estimate at Week 12 was positive with a 95% CI not crossing 0, suggesting an SD‐side advantage for this parameter. Hemoglobin variation, however, remained more variable across visits. These findings suggest a possible SD‐side advantage in hemoglobin‐related improvement at Week 12 after baseline adjustment, which may be relevant when vascular tone is a clinical consideration.

The most consistent and clinically salient between‐approach difference in this study was tolerability. Post‐procedure pain (VAS) was lower on the SD side at every treatment session, and embossing occurred only on the ID side, whereas it was not observed on the SD side. These findings have direct procedural relevance: SD injection may reduce immediate discomfort and visible transient surface irregularities, potentially improving patient acceptance and minimizing early post‐treatment aesthetic burden [25, 35, 37]. Global Aesthetic Improvement Scale (GAIS) ratings by both participants and investigators were high and did not demonstrate meaningful between‐side separation, supporting the interpretation that both planes can provide clinically appreciable improvement, with SD offering a more favorable immediate procedural profile. Although additional anesthetic strategies may reduce pain during ID injection, they entail additional procedural steps and do not directly address visible post‐injection embossing.

Several design features were incorporated to support interpretability of the findings. The split‐face, within‐subject design reduces inter‐individual variability, and ultrasound confirmation was used to verify placement in the intended injection plane. In addition, because tone‐related indices can be sensitive to baseline conditions, baseline‐adjusted mixed‐effects modeling was emphasized for outcomes showing baseline imbalance between sides, particularly hemoglobin‐related measures.

Despite these considerations, side‐related confounding cannot be fully excluded because allocation was fixed (left ID, right SD). Although the split‐face design reduced inter‐individual variability and baseline‐adjusted analyses were used where appropriate, unmeasured laterality‐related factors, such as inherent facial asymmetry, differential environmental exposure, habitual sleeping side, or operator‐handedness effects, may have influenced some outcomes. Future studies should consider randomized or counterbalanced side allocation to better isolate the effect of injection plane.

In addition, the sample size assumptions and the −15% non‐inferiority margin were based on clinical judgment, planning assumptions, general non‐inferiority principles, and precedent from aesthetic injectable/filler trials rather than a validated MCID for the Antera 3D fineline index; therefore, the primary finding should be interpreted as statistical non‐inferiority within the prespecified margin, and secondary outcomes should be regarded as exploratory because multiple endpoints were evaluated without multiplicity adjustment. Follow‐up was limited to Week 12, and the present study was not designed to evaluate longer‐term durability, product persistence, or cumulative effects with repeated treatments. Because injection depth may influence product distribution and tissue residence, longer‐term follow‐up is needed to determine whether ID and SD delivery differ in the persistence of skin‐quality improvement. Finally, although imaging‐derived indices provide objective quantification, they depend on standardized acquisition conditions and may exhibit measurement variability—particularly for tone‐related parameters—which should be considered when generalizing the findings.

In a split‐face comparison of intradermal versus subdermal injection of a polynucleotide‐based injectable medical device, subdermal delivery provided short‐term objective skin‐quality improvement comparable to intradermal delivery and demonstrated a more favorable immediate tolerability profile, with lower pain and no visible injection‐site embossing. These findings support considering subdermal injection as a practical option under the present standardized protocol when comparable short‐term skin‐quality improvement and reduced peri‐procedural burden are prioritized.

## Author Contributions

H.B.: Study design, supervision, statistical analysis, figure preparation, literature review and drafting of the manuscript; S.‐B.K., Y.‐R.H.: Ultrasonographic data acquisition, data analysis, and interpretation; H.J.P., J.S.K.: Data collection and management, literature review, and drafting of the introduction and discussion; N.‐K.R.: Conceptualization, study design, supervision, manuscript review and editing. H.‐J.K.: Study supervision, manuscript drafting, critical revision of the manuscript, and correspondence with the journal. All authors read and approved the final manuscript.

## Funding

This work was supported by PharmaResearch Co. Ltd, 2024‐31‐0667.

## Ethics Statement

This study was approved by the Public Institutional Review Board designated by the Ministry of Health and Welfare of the Republic of Korea (approval No. P01‐202404‐01‐049) and was conducted in accordance with the Declaration of Helsinki. Written informed consent was obtained from all participants prior to enrollment.

## Conflicts of Interest

The authors declare no conflicts of interest.

## Supporting information


**Figure S1:** Longitudinal changes in secondary objective skin‐quality measures (Weeks 0, 9, and 12).
**Figure S2:** Distributions of percentage improvement in secondary objective outcomes at Weeks 9 and 12.
**Figure S3:** GAIS outcomes rated by participants and investigators.

**Table S1:** Full eligibility criteria.
**Table S2:** Comparison of wrinkle index between intradermal and subdermal sides.
**Table S3:** Comparison of roughness index between intradermal and subdermal sides.
**Table S4:** Comparison of pore index between intradermal and subdermal sides.
**Table S5:** Comparison of melanin variation index between intradermal and subdermal sides.
**Table S6:** Comparison of hemoglobin index between intradermal and subdermal sides.
**Table S7:** Comparison of hemoglobin variation index between intradermal and subdermal sides.
**Table S8:** Comparison of skin elasticity (R2) index between intradermal and subdermal sides.
**Table S9:** Participant‐rated Global Aesthetic Improvement Scale (GAIS) at Weeks 9 and 12: intradermal versus subdermal injection.
**Table S10:** Investigator‐rated Global Aesthetic Improvement Scale (GAIS) at Weeks 9 and 12: intradermal versus subdermal injection.
**Table S11:** Injection‐site embossing severity after each treatment session (intradermal versus subdermal).

## Data Availability

The data that support the findings of this study are available on request from the corresponding author. The data are not publicly available due to privacy or ethical restrictions.

## References

[1] X. Dan , S. Li , H. Chen , et al., “Tailoring Biomaterials for Skin Anti‐Aging,” Materials Today Bio 28 (2024): 101210, 10.1016/j.mtbio.2024.101210. PMC11402947 39285945

[2] S. Hsin , K. Lourenço , A. Porcello , et al., “Clinical Safety and Efficacy of Hyaluronic Acid–Niacinamide–Tranexamic Acid Injectable Hydrogel for Multifactorial Facial Skin Quality Enhancement With Dark Skin Lightening,” Gels 11, no. 7 (2025): 495, 10.3390/gels11070495. 40710660 PMC12294899

[3] R. S. Hussein , S. Bin Dayel , O. Abahussein , and A. A. El‐Sherbiny , “Influences on Skin and Intrinsic Aging: Biological, Environmental, and Therapeutic Insights,” Journal of Cosmetic Dermatology 24, no. 2 (2025): e16688, 10.1111/jocd.16688. 39604792 PMC11845971

[4] W. P. Werschler , J. M. Calkin , D. A. Laub , T. Mauricio , V. A. Narurkar , and P. Rich , “Aesthetic Dermatologic Treatments: Consensus From the Experts,” Journal of Clinical and Aesthetic Dermatology 8, no. 10 Suppl (2015): S2–S7. PMC4635431 26587179

[5] H. Sumino , S. Ichikawa , M. Abe , Y. Endo , O. Ishikawa , and M. Kurabayashi , “Effects of Aging, Menopause, and Hormone Replacement Therapy on Forearm Skin Elasticity in Women,” Journal of the American Geriatrics Society 52, no. 6 (2004): 945–949, 10.1111/j.1532-5415.2004.52262.x. 15161459

[6] S. Humphrey , S. Manson Brown , S. J. Cross , and R. Mehta , “Defining Skin Quality: Clinical Relevance, Terminology, and Assessment,” Dermatologic Surgery 47, no. 7 (2021): 974–981, 10.1097/DSS.0000000000003079. 34148998 PMC8231670

[7] L. Devgan , P. Singh , and K. Durairaj , “Minimally Invasive Facial Cosmetic Procedures,” Otolaryngologic Clinics of North America 52, no. 3 (2019): 443–459, 10.1016/j.otc.2019.02.013. 30954270

[8] H. J. Kim , K. K. Seo , H. K. Lee , J. S. Kim , and K. H. Youn , Clinical Anatomy of the Face for Filler and Botulinum Toxin Injection (Springer Nature, 2024), 10.1007/978-981-99-7133-6.

[9] A. F. Klassen , C. Rae , L. Gallo , et al., “Measuring Satisfaction With Minimally Invasive Aesthetic Treatments With the SKIN‐Q Treatment Outcome Scale,” Aesthetic Surgery Journal 45, no. 9 (2025): 928–935, 10.1093/asj/sjaf075. 40392057 PMC12358213

[10] N. Kumar , D. Hye Suh , S. J. Lee , and H. J. Ryu , “Radiofrequency‐Based Treatments for Facial Rejuvenation: A Systematic Review of Efficacy, Safety, and Patient‐Centered Outcomes,” Aesthetic Surgery Journal Open Forum 7 (2025): ojaf159, 10.1093/asjof/ojaf159. 41426292 PMC12715571

[11] N. K. Rho , H. S. Kim , S. Y. Kim , and W. Lee , “Injectable “Skin Boosters” in Aging Skin Rejuvenation: A Current Overview,” Archives of Plastic Surgery 51, no. 6 (2024): 528–541, 10.1055/a-2366-3436. 39544509 PMC11560330

[12] J. H. Lee , J. Kim , Y. N. Lee , et al., “The Efficacy of Intradermal Hyaluronic Acid Filler as a Skin Quality Booster: A Prospective, Single‐Center, Single‐Arm Pilot Study,” Journal of Cosmetic Dermatology 23, no. 2 (2024): 409–416, 10.1111/jocd.15944. 37705328

[13] N. K. Roh , M. J. Kim , Y. W. Lee , Y. B. Choe , and K. J. Ahn , “A Split‐Face Study of the Effects of a Stabilized Hyaluronic Acid–Based Gel of Nonanimal Origin for Facial Skin Rejuvenation Using a Stamp‐Type Multineedle Injector: A Randomized Clinical Trial,” Plastic and Reconstructive Surgery 137, no. 3 (2016): 809–816, 10.1097/01.prs.0000480686.68275.60. 26910661

[14] W. G. Philipp‐Dormston , D. Bergfeld , B. M. Sommer , et al., “Consensus Statement on Prevention and Management of Adverse Effects Following Rejuvenation Procedures With Hyaluronic Acid‐Based Fillers,” Journal of the European Academy of Dermatology and Venereology: JEADV 31, no. 7 (2017): 1088–1095, 10.1111/jdv.14295. 28449190

[15] P. Trevidic , P. Andre , L. Benadiba , et al., “Prospective, Split‐Face, Randomized, Long‐Term Blinded Objective Comparison of the Performance and Tolerability of Two New Hyaluronic Acid Fillers,” Dermatologic Surgery 43, no. 12 (2017): 1448–1457, 10.1097/DSS.0000000000001193. 28595250

[16] I. T. Jones , M. J. Vanaman Wilson , J. Bolton , L. Zaleski‐Larsen , D. C. Wu , and M. P. Goldman , “A Single Center, Prospective, Randomized, Sham‐Controlled, Double‐Blinded, Split‐Face Trial Using Microinjections of Transparent Hyaluronic Acid Gel for Cheek Rejuvenation,” Dermatologic Surgery : Official Publication of the American Society for Dermatologic Surgery [Et Al.] 44, no. 6 (2018): 841–845, 10.1097/DSS.0000000000001460. 29381544

[17] Y. J. Lee , H. T. Kim , Y. J. Lee , et al., “Comparison of the Effects of Polynucleotide and Hyaluronic Acid Fillers on Periocular Rejuvenation: A Randomized, Double‐Blind, Split‐Face Trial,” Journal of Dermatological Treatment 33, no. 1 (2022): 254–260, 10.1080/09546634.2020.1748857. 32248707

[18] K. Beer , B. Biesman , S. E. Cox , S. Smith , L. Picault , and P. Trevidic , “Efficacy and Safety of Resilient Hyaluronic Acid Fillers Injected With a Cannula: A Randomized, Evaluator‐Blinded, Split‐Face Controlled Study,” Clinical, Cosmetic and Investigational Dermatology 16 (2023): 959–972, 10.2147/CCID.S402315. 37038451 PMC10082220

[19] J. H. Kim , E. S. Kim , S. W. Kim , S. P. Hong , and J. Kim , “Effects of Polynucleotide Dermal Filler in the Correction of Crow's Feet Using an Antera Three‐Dimensional Camera,” Aesthetic Plastic Surgery 46, no. 4 (2022): 1902–1909, 10.1007/s00266-022-02832-8. 35357558

[20] S. Lampridou , S. Bassett , M. Cavallini , and G. Christopoulos , “The Effectiveness of Polynucleotides in Esthetic Medicine: A Systematic Review,” Journal of Cosmetic Dermatology 24, no. 2 (2025): e16721, 10.1111/jocd.16721. 39645667 PMC11845969

[21] K. W. A. Lee , K. W. L. Chan , A. Lee , et al., “Polynucleotides in Aesthetic Medicine: A Review of Current Practices and Perceived Effectiveness,” International Journal of Molecular Sciences 25, no. 15 (2024): 8224, 10.3390/ijms25158224. 39125793 PMC11311621

[22] S. Y. Choi , Y. G. Koh , K. H. Yoo , H. S. Han , J. Seok , and B. J. Kim , “A Randomized, Participant‐ and Evaluator‐Blinded, Matched‐Pair, Prospective Study Comparing the Safety and Efficacy Between Polycaprolactone and Polynucleotide Fillers in the Correction of Crow's Feet,” Journal of Cosmetic Dermatology 24, no. 1 (2025): e16576, 10.1111/jocd.16576. 39313949 PMC11754925

[23] M. Cavallini , E. Bartoletti , L. Maioli , et al., “Consensus Report on the Use of PN‐HPT (Polynucleotides Highly Purified Technology) in Aesthetic Medicine,” Journal of Cosmetic Dermatology 20, no. 3 (2021): 922–928, 10.1111/jocd.13679. 32799391 PMC7984045

[24] N. K. Rho , S. Chilukuri , G. Chan , M. Kim , J. Shin , and A. Suwanchinda , “Expert Perspectives: Evidence‐Based Applications of Polynucleotides (PNs) in Aesthetic Medicine and Dermatology,” Clinical, Cosmetic and Investigational Dermatology 19 (2026): 1–9, 10.2147/CCID.S557226. PMC13047770 41939258

[25] J. Y. Hong , Y. H. Lee , H. Kim , and K. Y. Park , “Therapeutic Performance of Needle Injection Versus Needle‐Free Jet Injector System for Polynucleotide Filler in Skin Rejuvenation,” Journal of Cosmetic Dermatology 24, no. 1 (2025): e16595, 10.1111/jocd.16595. 39370844 PMC11743049

[26] C. Messaraa , A. Metois , M. Walsh , et al., “Antera 3D Capabilities for Pore Measurements,” Skin Research and Technology 24, no. 4 (2018): 606–613, 10.1111/srt.12472. 29707814

[27] C. Messaraa , A. Metois , M. Walsh , et al., “Wrinkle and Roughness Measurement by the Antera 3D and Its Application for Evaluation of Cosmetic Products,” Skin Research and Technology 24, no. 3 (2018): 359–366, 10.1111/srt.12436. 29368349

[28] A. R. Matias , M. Ferreira , P. Costa , and P. Neto , “Skin Colour, Skin Redness and Melanin Biometric Measurements: Comparison Study Between Antera 3D, Mexameter and Colorimeter,” Skin Research and Technology 21, no. 3 (2015): 346–362, 10.1111/srt.12199. 25645051

[29] F. Linming , H. Wei , L. Anqi , et al., “Comparison of Two Skin Imaging Analysis Instruments: The VISIA From Canfield vs the ANTERA 3DCS From Miravex,” Skin Research and Technology 24, no. 1 (2018): 3–8, 10.1111/srt.12381. 28585335

[30] D. Haykal , “3D Skin Mapping for Personalized Dermatological Treatment,” Frontiers in Photonics 6 (2025): 1535133, 10.3389/fphot.2025.1535133.

[31] S. Anqi , S. Xiukun , and X. Ai'e , “Quantitative Evaluation of Sensitive Skin by ANTERA 3D Combined With GPSkin Barrier,” Skin Research and Technology 28, no. 6 (2022): 840–845, 10.1111/srt.13213. 36308515 PMC9907598

[32] US Food and Drug Administration , Non‐Inferiority Clinical Trials to Establish Effectiveness (US Food Drug Adm, 2016).

[33] X. Dai , L. Li , W. Peterson , et al., “Safety and Effectiveness of Hyaluronic Acid Dermal Filler in Correction of Moderate‐To‐Severe Nasolabial Folds in Chinese Subjects,” Clinical, Cosmetic and Investigational Dermatology 12 (2019): 57–62. 30666143 10.2147/CCID.S187079 PMC6336025

[34] N. Froget , I. Kobylińska , O. Warszawik‐Hendzel , T. Guidicelli , and J. Gotlib , “Effectiveness and Safety of IPN‐20‐SENSE LIDOCAINE for Lip Volume Augmentation and/or Redefinition (SMILE Study): A Non‐Inferiority Randomized Double‐Blinded Controlled Study,” Aesthetic Plastic Surgery 49, no. 4 (2025): 1033–1045. 39402200 10.1007/s00266-024-04398-z PMC11893706

[35] T. S. Lim , S. Liew , X. J. Tee , et al., “Polynucleotides HPT for Asian Skin Regeneration and Rejuvenation,” Clinical, Cosmetic and Investigational Dermatology 17 (2024): 417–431, 10.2147/CCID.S437942. 38371328 PMC10874187

[36] Y. Morihisa , Y. Rikimaru‐Nishi , Y. Ohmaru , K. Ino , H. Rikimaru , and K. Kiyokawa , “Scientific Validation of Clinical Visual Scales and Antera 3D Consistency With Derived Measurements in the Assessment of Infantile Haemangioma After Laser Therapy,” Journal of Plastic, Reconstructive & Aesthetic Surgery 91 (2024): 47–55, 10.1016/j.bjps.2024.01.019. 38401278

[37] L. Shi , J. Zhang , G. Wang , et al., “A Cross‐Sectional Survey on Pain Management in Dermal Filler Injections From Physicians' and Patients' Perspectives,” Aesthetic Plastic Surgery 48, no. 7 (2024): 1417–1425, 10.1007/s00266-023-03843-9. 38305924

