# Correction to “Efficacy and Safety of Intradermal Versus Subdermal Injection of Polynucleotide for Facial Improvement: A Prospective Split‐Face Study”

**DOI:** 10.1111/jocd.71242

**Published:** 2026-11-08

Bae H , Kim S‐B , Heo Y‐R , Park HJ , Kim JS , Rho N‐K , and Kim H‐J , “Efficacy and Safety of Intradermal Versus Subdermal Injection of Polynucleotide for Facial Improvement: A Prospective Split‐Face Study,” Journal of Cosmetic Dermatology 25, (2026): e71108, 10.1111/jocd.71108.

In the originally published article, the Conflict of Interest statement was incomplete. The statement “The authors declare no conflicts of interest.” should have read:

“Nark‐Kyoung Rho and Hyoun Jun Park serve as advisory board members for PharmaResearch Co. Ltd., providing consultation on general company policy. These advisory activities are separate from the present study, and neither received personal research funding, study‐related honoraria, or other personal compensation in connection with this study. The other authors declare no conflicts of interest.”

We apologize for this error.

