# Supplementary material for: Efficacy and Safety of Intradermal Versus Subdermal Injection of Polynucleotide for Facial Improvement: A Prospective Split‐Face Study
Source: J Cosmet Dermatol. 2026 Aug 4;25(8):e71108. doi: 10.1111/jocd.71108 (PMC13434674; doi:10.1111/jocd.71108)
Supplement: Supplementary file 1 [file JOCD-25-e71108-s001.docx]

**Figure S1. Longitudinal changes in secondary objective skin-quality measures (Weeks 0, 9, and 12).**

Mean values (±SEM) are shown for the intradermal (ID) and subdermal (SD) sides at baseline, Week 9, and Week 12 for wrinkle index, roughness index, pore index, melanin variation, hemoglobin index, hemoglobin variation, and skin elasticity (R2). Hemoglobin-related measures and skin elasticity are presented as baseline-adjusted model-estimated means derived from linear mixed-effects modeling with baseline as a covariate and subject as a random effect. Asterisks indicate within-side differences versus baseline (*p<0.05, **p<0.01, ***p<0.001); ns indicates not significant.

**Figure S2. Distributions of percentage improvement in secondary objective outcomes at Weeks 9 and 12.**

Box plots show the distribution of percent improvement from baseline for the intradermal (ID) and subdermal (SD) sides at Weeks 9 and 12 for wrinkle index, roughness, pore parameter, melanin variation, hemoglobin index, hemoglobin variation, and skin elasticity (R2). Boxes indicate the interquartile range with the median; whiskers indicate the range (excluding outliers, if applicable), and individual participant values are overlaid as points. Percent improvement was calculated relative to each side’s baseline value.

**Figure S3. GAIS outcomes rated by participants and investigators.**

Global Aesthetic Improvement Scale (GAIS) distributions and responder rates (GAIS ≥1) are shown for the intradermal (ID; left cheek) and subdermal (SD; right cheek) sides at Week 9 and Week 12. The left panels display the percentage distribution of GAIS categories (very much improved, much improved, improved, no change, worse). The right panels show the overall improvement rate, defined as the proportion of subjects with GAIS ≥1 at each time point. GAIS results are presented descriptively (exploratory).
