# Supplementary material for: Efficacy and Safety of Intradermal Versus Subdermal Injection of Polynucleotide for Facial Improvement: A Prospective Split‐Face Study
Source: J Cosmet Dermatol. 2026 Aug 4;25(8):e71108. doi: 10.1111/jocd.71108 (PMC13434674; doi:10.1111/jocd.71108)
Supplement: Supplementary file 2 [file JOCD-25-e71108-s002.docx]

**Table S1. Full eligibility criteria.**

| **Inclusion criteria (all required)** | **Exclusion criteria (any criterion met)** | **Withdrawal/Discontinuation criteria** |
| --- | --- | --- |
| Age ≥19 years (men or women). | Received facial (cheek) filler injection within the previous 6 months. | Withdrawal of consent (participant or legal representative) |
| Seeking improvement of facial wrinkles. | Currently receiving NSAIDs, antiplatelet agents, anticoagulants, or immunosuppressants. | Ineligibility identified after enrollment |
| Agreed to discontinue all dermatologic/aesthetic procedures or treatments for facial wrinkle improvement during the study period. | Had allergic disease, autoimmune disease, sarcoid granulomatous pathology, or Osler’s endocarditis. | Adverse events precluding continuation |
| Provided voluntary written informed consent after receiving a full explanation of the study. | Known hypersensitivity to the investigational device component (sodium polynucleotide). | Inability to complete follow-up. |
|  | Pregnant or breastfeeding. | Pregnancy during the study period. |
|  | Lacked capacity for understanding and/or communication adequate for study participation. | Other investigator-determined reasons for discontinuation |
|  | Had damaged skin at the treatment sites, including inflammation or infection. |  |

**Table S2. Comparison of wrinkle index between intradermal and subdermal sides.**

|  |  | **Intradermal** | **Subdermal** | **p-value** |
| --- | --- | --- | --- | --- |
| **Baseline** | N | 37 | 37 | **0.824^#a^** |
|  | Mean±SD | 9.71±1.75 | 9.66±2.23 |  |
|  | [Min–Max] | [6.81–13.21] | [6.75–14.91] |  |
| **Week 9** | N | 37 | 37 |  |
|  | Mean±SD | 8.88±1.28 | 8.63±1.78 |  |
|  | [Min–Max] | [6.22–11.03] | [5.56–12.51] |  |
|  | p-value | **<0.001^#b^** | **<0.001^†b^** | **0.308^#a^** |
| **Week 12** | N | 37 | 37 |  |
|  | Mean±SD | 9.27±1.58 | 8.89±1.74 |  |
|  | [Min–Max] | [6.84–12.81] | [5.84–14.17] |  |
|  | p-value | **0.003^#b^** | **<0.001^†b^** | **0.099^#a^** |

*Values are presented as Mean ± Standard Deviation and [Minimum – Maximum]. a: Between-side comparison between ID and SD. b: Within-side comparison versus baseline (0W). #: Analyzed by Paired t-test. †: Analyzed by Wilcoxon signed-rank test (normality not met).* ***ID****, intradermal;* ***SD****, subdermal.*

**Table S3. Comparison of roughness index between intradermal and subdermal sides.**

|  |  | **Intradermal** | **Subdermal** | **p-value** |
| --- | --- | --- | --- | --- |
| **Baseline** | N | 37 | 37 | **0.794^#a^** |
|  | Mean±SD | 9.68±1.88 | 9.61±2.38 |  |
|  | [Min–Max] | [6.41–13.41] | [6.75–14.91] |  |
| **Week 9** | N | 37 | 37 |  |
|  | Mean±SD | 8.80±1.72 | 8.46±1.85 |  |
|  | [Min–Max] | [5.95– 12.22] | [5.51–12.80] |  |
|  | p-value | **<0.001^#b^** | **<0.001^#b^** | **0.156^#a^** |
| **Week 12** | N | 37 | 37 |  |
|  | Mean±SD | 9.09±1.81 | 8.86±1.88 |  |
|  | [Min–Max] | [6.22–13.02] | [5.77–14.74] |  |
|  | p-value | **<0.001^#b^** | **<0.001^#b^** | **0.531^†a^** |

*Values are presented as Mean ± Standard Deviation and [Minimum – Maximum]. a: Between-side comparison between ID and SD. b: Within-side comparison versus baseline (0W). #: Analyzed by Paired t-test. †: Analyzed by Wilcoxon signed-rank test (normality not met).* ***ID****, intradermal;* ***SD****, subdermal.*

**Table S4. Comparison of pore index between intradermal and subdermal sides.**

|  |  | **Intradermal** | **Subdermal** | **p-value** |
| --- | --- | --- | --- | --- |
| **Baseline** | N | 37 | 37 | **0.803^†a^** |
|  | Mean±SD | 0.30±0.13 | 0.31±0.16 |  |
|  | [Min–Max] | [0.10–0.68] | [0.08–0.75] |  |
| **Week 9** | N | 37 | 37 |  |
|  | Mean±SD | 0.23±0.09 | 0.24±0.13 |  |
|  | [Min–Max] | [0.10– 0.51] | [0.06–0.66] |  |
|  | p-value | **<0.001^#b^** | **<0.001^†b^** | **0.740^†a^** |
| **Week 12** | N | 37 | 37 |  |
|  | Mean±SD | 0.27±0.11 | 0.27±0.16 |  |
|  | [Min–Max] | [0.08–0.60] | [0.00–0.76] |  |
|  | p-value | **0.079^†b^** | **0.095^#b^** | **0.892^†a^** |

*Values are presented as Mean ± Standard Deviation and [Minimum – Maximum]. a: Between-side comparison between ID and SD. b: Within-side comparison versus baseline (0W). #: Analyzed by Paired t-test. †: Analyzed by Wilcoxon signed-rank test (normality not met).* ***ID****, intradermal;* ***SD****, subdermal.*

**Table S5. Comparison of melanin variation index between intradermal and subdermal sides.**

|  |  | **Intradermal** | **Subdermal** | **p-value** |
| --- | --- | --- | --- | --- |
| **Baseline** | N | 37 | 37 | **0.311^†a^** |
|  | Mean±SD | 0.035±0.008 | 0.035±0.010 |  |
|  | [Min–Max] | [0.021–0.051] | [0.023–0.060] |  |
| **Week 9** | N | 37 | 37 |  |
|  | Mean±SD | 0.034±0.010 | 0.033±0.010 |  |
|  | [Min–Max] | [0.015–0.057] | [0.018–0.060] |  |
|  | p-value | **0.114^†b^** | **0.105^†b^** | **0.316^†a^** |
| **Week 12** | N | 37 | 37 |  |
|  | Mean±SD | 0.035±0.009 | 0.032±0.008 |  |
|  | [Min–Max] | [0.016–0.053] | [0.020–0.048] |  |
|  | p-value | **0.154^†b^** | **0.019^†b^** | **0.041^#a^** |

*Values are presented as Mean ± Standard Deviation and [Minimum – Maximum]. a: Between-side comparison between ID and SD. b: Within-side comparison versus baseline (0W). #: Analyzed by Paired t-test. †: Analyzed by Wilcoxon signed-rank test (normality not met).* ***ID****, intradermal;* ***SD****, subdermal.*

**Table S6. Comparison of hemoglobin index between intradermal and subdermal sides.**

|  |  | **Intradermal** | **Subdermal** | **p-value** |
| --- | --- | --- | --- | --- |
| **Baseline** | N | 37 | 37 | **<0.001^#a^** |
|  | Mean±SD | 1.34±0.17 | 1.26±0.18 |  |
|  | [Min–Max] | [1.01–1.75] | [0.93–1.78] |  |
| **Week 9** | N | 37 | 37 |  |
|  | Mean±SD | 1.22±0.25 | 1.17±0.15 |  |
|  | [Min–Max] | [0.11–1.64] | [0.84–1.57] |  |
|  | p-value | **<0.001^†b^** | **<0.001^#b^** | **<0.001^†a^** |
| **Week 12** | N | 37 | 37 |  |
|  | Mean±SD | 1.29±0.14 | 1.19±0.13 |  |
|  | [Min–Max] | [0.98–1.52] | [0.89–1.43] |  |
|  | p-value | **<0.01^†b^** | **<0.001^†b^** | **<0.001^#a^** |

*Values are presented as Mean ± Standard Deviation and [Minimum – Maximum]. a: Between-side comparison between ID and SD. b: Within-side comparison versus baseline (0W). #: Analyzed by Paired t-test. †: Analyzed by Wilcoxon signed-rank test (normality not met).* ***ID****, intradermal;* ***SD****, subdermal.*

**Table S7. Comparison of hemoglobin variation index between intradermal and subdermal sides.**

|  |  | **Intradermal** | **Subdermal** | **p-value** |
| --- | --- | --- | --- | --- |
| **Baseline** | N | 37 | 37 | **<0.001^#a^** |
|  | Mean±SD | 0.087±0.020 | 0.104±0.032 |  |
|  | [Min–Max] | [0.054–0.133] | [0.054–0.172] |  |
| **Week 9** | N | 37 | 37 |  |
|  | Mean±SD | 0.078±0.017 | 0.091±0.030 |  |
|  | [Min–Max] | [0.042–0.130] | [0.054–0.182] |  |
|  | p-value | **<0.01^#b^** | **<0.001^#b^** | **0.057^†a^** |
| **Week 12** | N | 37 | 37 |  |
|  | Mean±SD | 0.082±0.019 | 0.094±0.025 |  |
|  | [Min–Max] | [0.049–0.123] | [0.050–0.158] |  |
|  | p-value | **<0.01^†b^** | **<0.001^#b^** | **<0.05^#a^** |

*Values are presented as Mean ± Standard Deviation and [Minimum – Maximum]. a: Between-side comparison between ID and SD. b: Within-side comparison versus baseline (0W). #: Analyzed by Paired t-test. †: Analyzed by Wilcoxon signed-rank test (normality not met).* ***ID****, intradermal;* ***SD****, subdermal.*

**Table S8. Comparison of skin elasticity (R2) index between intradermal and subdermal sides.**

|  |  | **Intradermal** | **Subdermal** | **p-value** |
| --- | --- | --- | --- | --- |
| **Baseline** | N | 37 | 37 | **0.069^#a^** |
|  | Mean±SD | 0.695±0.080 | 0.679±0.093 |  |
|  | [Min–Max] | [0.552–0.868] | [0.504–0.893] |  |
| **Week 9** | N | 37 | 37 |  |
|  | Mean±SD | 0.741±0.099 | 0.729 ±0.101 |  |
|  | [Min–Max] | [0.541–0.886] | [0.507–0.968] |  |
|  | p-value | **<0.05^#b^** | **<0.05^#b^** | **0.078^†a^** |
| **Week 12** | N | 37 | 37 |  |
|  | Mean±SD | 0.737±0.096 | 0.696±0.076 |  |
|  | [Min–Max] | [0.552–0.925] | [0.537–0.900] |  |
|  | p-value | **<0.05^#b^** | **0.353^#b^** | **<0.05^#a^** |

*Values are presented as Mean ± Standard Deviation and [Minimum – Maximum]. a: Between-side comparison between ID and SD. b: Within-side comparison versus baseline (0W). #: Analyzed by Paired t-test. †: Analyzed by Wilcoxon signed-rank test (normality not met).* ***ID****, intradermal;* ***SD****, subdermal.*

**Table S9. Participant-rated Global Aesthetic Improvement Scale (GAIS) at Weeks 9 and 12: intradermal versus subdermal injection.**

|  | **Intradermal** | **Subdermal** |
| --- | --- | --- |
| **Week 9, n(%)** |  |  |
| Very much improved | 6 (16.2) | 7 (18.9) |
| Much improved | 17 (45.9) | 17 (45.9) |
| Improved | 12 (32.4) | 10 (27.0) |
| No change | 2 (5.4) | 3 (8.1) |
| Worse | 0 (0.0) | 0 (0.0) |
| **Improved subjects*^b^*** | 35 (94.6) | 34 (91.9) |
| P-value for GAIS ≥1 | **0.928^c^** |  |
| **Week 12, n(%)** |  |  |
| Very much improved | 6 (16.2) | 6 (16.2) |
| Much improved | 11 (29.7) | 13 (35.1) |
| Improved | 12 (32.4) | 11 (29.7) |
| No change | 7 (18.9) | 7 (18.9) |
| Worse | 1 (2.7) | 0 (0.0) |
| **Improved subjects*^b^*** | 29 (78.3) | 30 (81.1) |
| P-value for GAIS ≥1 | **0.876^c^** |  |

*Values are presented as n (%). ^b^: “Improved subject” includes participants rated as “improved,” “much improved,” or “very much improved” (GAIS ≥1). ^c^: p values are exploratory/descriptive and were calculated for the binary comparison of GAIS ≥1 vs <1 between ID and SD using Pearson’s chi-square test (or Fisher’s exact test when appropriate). ID, intradermal; SD, subdermal; GAIS, Global Aesthetic Improvement Scale.*

**Table S10. Investigator-rated Global Aesthetic Improvement Scale (GAIS) at Weeks 9 and 12: intradermal versus subdermal injection.**

|  | **Intradermal** | **Subdermal** |
| --- | --- | --- |
| **Week 9, n(%)** |  |  |
| Very much improved | 8 (21.6) | 7 (18.9) |
| Much improved | 21 (56.7) | 18 (48.6) |
| Improved | 6 (16.2) | 11 (29.7) |
| No change | 1 (2.7) | 0 (0.0) |
| Worse | 1 (2.7) | 1 (2.7) |
| **Improved subjects*^b^*** | 35 (94.5) | 36 (97.3) |
| P-value for GAIS ≥1 | **0.597^c^** |  |
| **Week 12, n(%)** |  |  |
| Very much improved | 10 (27.0) | 12 (32.4) |
| Much improved | 20 (54.1) | 15 (40.5) |
| Improved | 6 (16.2) | 8 (21.6) |
| No change | 0 (0.0) | 0 (0.0) |
| Worse | 1 (2.7) | 2 (5.4) |
| **Improved subjects*^b^*** | 36 (97.3) | 35 (94.6) |
| P-value for GAIS ≥1 | **0.679^c^** |  |

*Values are presented as n (%). ^b^: “Improved subject” includes participants rated as “improved,” “much improved,” or “very much improved” (GAIS ≥1). ^c^: p values are exploratory/descriptive and were calculated for the binary comparison of GAIS ≥1 vs <1 between ID and SD using Pearson’s chi-square test (or Fisher’s exact test when appropriate). ID, intradermal; SD, subdermal; GAIS, Global Aesthetic Improvement Scale.*

**Table S11. Injection-site embossing severity after each treatment session (intradermal versus subdermal).**

|  |  | **1^st^ treatment** | **2^nd^ treatment** | **3^rd^ treatment** |
| --- | --- | --- | --- | --- |
| **Intradermal n(%)** | Mild | 16(43.2) | 18(48.6) | 14(37.8) |
|  | Moderate | 21(56.8) | 18(48.6) | 21(56.8) |
|  | Severe | 0 | 1(2.7) | 2(5.4) |
| **Subdermal n(%)** | Mild | 0 | 0 | 0 |
|  | Moderate | 0 | 0 | 0 |
|  | Severe | 0 | 0 | 0 |

*Values are presented as n (%) based on participants with available assessments at each session.
Embossing was assessed within 10 minutes after injection and graded as none, mild, moderate, or severe; the “none” category is not shown in the table.
Subdermal injections showed no embossing events at any session (all categories 0). Abbreviations: ID, intradermal; SD, subdermal.*
